# Nurses' Views on Barriers to Oral Care in Non‐Mechanically Ventilated Patients in the Intensive Care Unit: A Qualitative Study

**DOI:** 10.1111/nicc.70173

**Published:** 2025-09-11

**Authors:** Auxillia Madhuvu, Marilyn Andrews, Carly Rienecker, Ashilta Prasad, Wendy Pollock

**Affiliations:** ^1^ School of Nursing and Midwifery, Monash University Frankston Victoria Australia; ^2^ Monash Health Dandenong Victoria Australia; ^3^ Peninsula Health Frankston Victoria Australia

**Keywords:** critical care nurses, hospital‐associated pneumonia, intensive care, intensive care unit, nurse, oral care, qualitative

## Abstract

**Background:**

Optimal oral care is essential in preventing non‐ventilator hospital‐associated pneumonia and enhancing patient comfort. However, nurses' clinical oral care practices for patients not on mechanical ventilation in the intensive care unit are both underreported and understudied.

**Aim:**

To explore intensive care nurses' clinical oral care practices for patients not on mechanical ventilation in intensive care units.

**Study Design:**

A qualitative descriptive study using semi‐structured in‐depth individual interviews. Intensive care nurses with more than 6 months experience were recruited via the professional association, the Australian College of Critical Care Nurses and snowballing. Purposive sampling was used, and information power was used to indicate when to stop data collection. Interviews were undertaken with ten intensive care nurses working with adult patients between May and September 2023. The interviews were audio‐recorded, transcribed and analysed using Framework Analysis.

**Results:**

Most of the participants had a postgraduate qualification in intensive care nursing. Four main themes were derived from the data: (i) challenging patient needs and prioritisation of care, (ii) inadequate knowledge and education, (iii) perception of inadequate use of evidence‐based practice and equipment and (iv) structured support needed for evidence‐based practice.

**Conclusions:**

These themes highlight ongoing issues with nurses' knowledge and education and implementation of available evidence‐based practice. Findings suggest the need for a well‐established policy to underpin practice. The barriers faced by intensive care nurses need to be addressed to enhance the quality of patient care.

**Relevance to Clinical Practice:**

Improving oral care in non‐mechanically ventilated patients would decrease the rates of hospital‐associated pneumonia, systemic diseases such as respiratory and cardiovascular diseases, and promote patient comfort. A comprehensive strategy using an implementation framework is required to address nurses' knowledge gaps and techniques that enhance evidence‐based practices.

**Impact Statements**

- What is known about the topic
  ○ Provision of oral care by intensive care nurses is essential.
  ○ Oral care helps to prevent HAP.
  ○ There is currently limited information on HAP prevention in non‐mechanically ventilated patients in ICU.
- What this paper adds
  ○ Provides perspectives of barriers to oral care practices across various organisations in Australian ICUs.
  ○ Highlights the lack of progress in oral care practices as an intervention to prevent NV‐HAP
  ○ Nurses reported a lack of institutional policy and procedures for oral care; an institutional discussion focused on NV‐HAP prevention and oral care should be considered.

## 1 Introduction

Oral health is a form of preventive healthcare practice that involves maintaining a clean mouth and teeth, promoting overall well‐being, comfort, and confidence of an individual. Many patients are admitted to hospital with bacteria colonised oral cavity which potentially worsens with time [1]. Patients in intensive care are critically ill, nil orally, have a feeling of thirst, and are vulnerable due to the severity of their sickness [2, 3]. According to an observational study in the critical care unit (CCU) by Needleman and colleagues, non‐mechanically ventilated patients' oral health deteriorated following admission to CCU due to medical conditions or interventions that negatively affect oral health, including patients dependent on nursing staff due to medical conditions and severity of sickness [4]. The oral microbiome of patients in the intensive care unit (ICU) is comprised of billions of bacteria, some of which may be biomarkers of specific diseases [5, 6]. The makeup of the oral microbiome is associated with a higher risk of oral diseases, reduced quality of life, and a greater likelihood of healthcare‐associated infections (HAIs), including healthcare‐associated pneumonia (NV‐HAP) [4, 7].

## 2 Background

Healthcare‐associated infections (HAI) are acquired as a direct or indirect result of healthcare and are a major burden to public health [8]. Non‐ventilator hospital‐associated pneumonia (NV‐HAP) is the most common HAI and accounts for approximately 30% of all HAIs [9, 10]. NV‐HAP is associated with increased length of stay in hospital, increased morbidity, mortality and healthcare costs [11, 12, 13]. Patients with NV‐HAP are eight times more likely to die in hospital than similar patients without NV‐HAP [13].

Hospital respiratory pathogens are more commonly found in the mouths of those who are unable to clear their secretions [14, 15]. NV‐HAP occurs when a patient aspirates their own oropharyngeal material, causing an inflammatory response [15]. NV‐HAP is also associated with prior oral carriage of common hospital pathogens [16] which reinforces the need for improvements in oral care. HAI are not inevitable, and prevention programmes such as the use of evidence‐based prevention bundles to optimise adherence have been successful at reducing the incidence. Evidence suggests that improving oral care may reduce the incidence of NV‐HAP [17]. Reducing colonisation of the mouth from hospital pathogens improves patient oral hygiene and decreases the risk of NV‐HAP [18, 19]. However, oral care is currently not performed optimally in the hospital environment [3]. A recent integrated review reported that there is an existing gap in the literature on oral care in non‐mechanically ventilated patients in ICU [3]. Most of the research has focused on nurses' knowledge of ventilator‐associated pneumonia (VAP), which is mechanical ventilation device‐associated pneumonia that develops when a patient is intubated and ventilated for more than 48 h [20]. However, an increase in non‐mechanically ventilated critically ill patients in ICU has been reported in low, medium and high‐income countries [21, 22]. Intensive care nurses play a pivotal role in the prevention and management of NV‐HAP as they are involved with hygienic care, observations and monitoring of the patient. However, there is little research on intensive care nurses' clinical oral care practices in patients not receiving mechanical ventilation. This study focused on the ICU setting because most of the patients are critically ill and at higher risk of developing HAI. Additionally, there are studies that have reported that oral care was sub‐optimal in hospitalised patients [3, 23]; thus, understanding intensive care nurses' clinical oral care practices, barriers and enablers in non‐mechanically ventilated patients is important.

### 2.1 Aim and Objectives of Study

The aim of this study was to explore intensive care nurses' clinical oral care practices for patients in ICU who were not on mechanical ventilation.

The objectives of this study were to:

- Explore intensive care nurses' clinical oral care practices in non‐ventilated patients in ICU.
- Identify enablers and barriers to the provision of optimal oral care.

### 2.2 Design and Methods

A qualitative descriptive design was used according to Sandelowski [24], with an inductive approach to explore nurses' views and beliefs of barriers and enablers to oral care in non‐mechanically ventilated patients in ICU. This study design enabled the researchers to explore the phenomena in‐depth and gather rich data [25]. Consolidated criteria for reporting qualitative research (COREQ) guidelines were followed [26].

#### 2.2.1 Setting and Sample

The study was conducted in Australia, with registered nurses (RN) who were working clinically in ICU. A purposive sample of RNs working in Australian ICUs, with more than 6 months experience caring for critically ill non‐ventilated patients, were recruited for this study. The participants were from different Australian States and Territories, different experience and education levels, RN with and without postgraduate ICU nursing qualifications, public and private ICUs. Exclusion criteria included professionals who were not RNs and those who were working in roles without direct patient contact. The information power approach and purposive sampling provided guidance on the sample size of ten participants [27]. All the participants had relevant knowledge, experience and insight in the topic.

An invitation to participate in the study was distributed to members of the Australian College of Critical Care Nurses (ACCCN) and via social media channels. ACCCN is a professional association for critical care nurses, and it has members in every Australian State and Territory [28]. The advertisement email included a QR code with a participant information sheet and a screening survey using the Qualtrics survey platform. The screening survey ensured that only eligible nurses proceeded for interview consideration, and it was clearly stated in the participant information sheet that not all intensive care nurses who volunteered to be interviewed would be interviewed, as researchers were following a specific sampling strategy.

#### 2.2.2 Data Collection Tools and Methods

Semi‐structured interviews were conducted with intensive care nurses via video calling at a mutually convenient time. The semi‐structured interview questions were designed with the research team, which consists of registered nurses currently working in ICU (Supplementary file 1). The interview questions covered: (i) nurses' clinical oral care practices, and (ii) enablers and barriers to oral care and NV‐HAP prevention. The participants' demographic characteristics, such as years of experience and role in ICU, were collected before the interview was scheduled via the recruitment screening survey. Memo notes were written during and after the interview and were used to inform decisions on further interviews. The interviews were conducted and audio‐recorded by two ICU nurses who are both experienced researchers (AM and WP). The audio‐recorded data were transcribed by a professional transcriber, and the data were analysed by the research team. The data were analysed concurrently with data collection.

#### 2.2.3 Data Analysis

The transcribed data were imported into Nvivo, a qualitative data software. The data were thematically analysed using the Framework Method to identify themes and patterns in the data [29, 30]. The transcripts were analysed separately, with coded phrases organised, sorted, and compared across multiple transcripts. The data were read and re‐read to capture an overarching impression, with initial reflections noted as brief memos. Coded phrases were subsequently abstracted into categories, which were further refined into an analytical framework. Discrepancies in coding were discussed before agreement on the main themes that were applied consistently throughout the whole dataset. Throughout this process, all analysis steps and identified themes were examined concurrently and independently by three (AM, CR and WP) research group members to enhance reliability. Then the entire research team met and discussed the codes and themes for further refinement. Quotations with nurses' role were used in presenting the findings to allow the reader to judge the trustworthiness of the interpretations.

### 2.3 Ethical and Research Approvals

A low/negligible risk ethics application was submitted to the Human Research Ethics Committee and approved (Project ID: 37366) on March 7, 2023. All participants were provided with a plain language statement that clearly outlined the study intentions. Participants consented to the study verbally and in writing prior to the interview. Only the researchers had access to the audio‐recordings and transcripts. Data were managed securely in keeping with the Australian National Statement on Ethical Conduct in Human Research 2023 [31].

#### 2.3.1 Rigour and Reflexivity

All team members have a common assumption that oral health care is a fundamental part of nursing care and can make a difference to patient outcomes. All researchers were aware of their theoretical and philosophical orientations and were reflexive throughout the research to avoid influencing the findings. Their goal was to present findings that accurately represent the views of the participants.

To ensure trustworthiness throughout the research process, several strategies were implemented. Confirmability was maintained by keeping an audit trail of coding decisions and practicing researcher reflexivity. Credibility was achieved by being open to all potential themes, carefully analysing negative cases, and having independent data analysis by research team members. Data reduction and conclusion‐drawing were supported by thorough verification with the data. In disagreements about coding decisions or key categories, the first and last authors discussed interpretations until they reached a consensus [29, 30]. Dependability was ensured through detailed methodological descriptions, and transferability was achieved through rich, thick descriptions of the data.

## 3 Findings

The ten participants in this study were nurses working in Australian ICUs, with interviews undertaken between May and September 2023. The interviews ranged in length from 40 to 55 min. The average interview time was 42.4 min. The participants had a range of characteristics, experience and education (Table 1). The majority of the participants (*n* = 9, 90%) had a postgraduate qualification in intensive care nursing and were working clinically as bedside nurses, access nurses or in charge of the shift. The senior nurses were clinical nurse consultant/manager (CNC), clinical nurse specialist (CNS) and the junior nurses were critical care registered nurses (CCRN) and registered nurses (RNs). Data excerpts are reported by participants' role, number of the interview and state, for example, Senior Nurse 7_SA.

**TABLE 1:** Participant characteristics.

	(*N =* 10)
	*n* (%)
Age	
< 30	1 (10)
31–40	4 (40)
41–50	4 (40)
> 50	1 (10)
*Years of experience*	
< 5	3 (30)
5–10	1 (10)
> 10	6 (60)
*Post graduate qualification*	
Certificate	3 (30)
Diploma	4 (40)
Masters	2 (20)
None	1 (20)
*Position in ICU*	
CCRN	3 (30)
CNS	5 (50)
CNC/Manager	1 (10)
RN	1 (10)
*State*	
ACT	2 (20)
NSW	2 (20)
SA	1 (10)
TAS	1 (10)
VIC	3 (30)
WA	1 (10)

Abbreviations: ACT, Australian Capital Territory; CCRN, Critical Care Registered Nurse; Certificate, Cert; CNC, Clinical Nurse Consultant; CNS, Clinical Nurse Specialist; Diploma, Dip; Masters, MN; NSW, New South Wales; RN, Registered Nurse; SA, South Australia; TAS, Tasmania; VIC, Victoria; WA, Western Australia.

Four themes were identified from the data: (i) challenging patient needs and prioritisation of care, (ii) inadequate knowledge and education, (iii) perception of inadequate use of evidence‐based practice and equipment and (iv) structured support needed for evidence‐based practice. Each theme consisted of various subthemes (Table 2).

**TABLE 2:** Themes and subthemes.

Themes	Subthemes
Challenging patient needs and prioritisation of care	Patient condition
	Competing patient needs
	Cultural awareness
Inadequate knowledge and education	Lack of current knowledge and educational preparedness
Perception of inadequate use of evidence‐based practice and equipment	Lack of policy or procedures
	Lack of consensus on preferred approaches and tools
Structured support needed for evidence‐based practice	Leadership support (supervision, provision of resources)
	Ongoing nurse and patient education (in‐services, bed‐side; post‐graduate)
	Create awareness of the importance of oral care (visual reminders)

### 3.1 Challenging Patient Needs and Prioritisation of Care

Most of the participants described the patients they were providing care to as: unstable, confused and agitated at times and their experience caring for them and providing oral care as challenging and very demanding. The participants reported the importance of prioritising patient care according to the patient's condition at a given time as illustrated by the following subthemes: patient condition, competing care needs and cultural considerations.

#### 3.1.1 Patient Condition

Patient's neurological status was determined when the participants' provided oral care. Some participants in this study reported situations when they could not perform oral care as the patient was agitated and it was unsafe for the patient and staff to provide such care:

—the patients are like severely delirious or intermittently agitated or restless, they're not able to follow commands, and you're unable to approach patient to perform certain tasks. (Junior Nurse 6_NSW)

Some patients missed on oral care because they were reported to be unstable on non‐invasive ventilation continuously, with the face mask covering the mouth.

#### 3.1.2 Competing Patient Needs

Some participants reported that the priority of care was given to haemodynamic monitoring, patient safety and medical procedures such as scans, while oral care was prioritised lower. Some participants reported prioritising other nursing care activities, such as pressure area care and medications, over oral care. Patient rest periods were also prioritised in situations when patients were agitated and eventually got settled:

*…*sometimes you don't want to poke the bear. So, if they are finally settled you don't want to go poking swabs or toothbrushes in their mouths or trying to suction their mouth. (Junior Nurse 2_VIC)

However, some participants reported that the lack of awareness of NV‐HAP determines the nurse's attitude and their prioritisation of fundamental nursing care:

–a perception out there that it's not ‐ not needed, but it's not as important as other things. And medications come first. Obviously, ABC [airway, breathing, circulation] comes first, but medications, mobilising someone, all come first. But I think maybe lack of prioritisation or awareness. (Senior Nurse 5_NSW)

#### 3.1.3 Cultural Awareness

Some participants highlighted situations where patients depended heavily on caregivers, expecting registered nurses to handle every aspect of their care, irrespective of their ability to perform the activity themselves due to their cultural orientation:

That's just how things work in his culture; he would sort of demand the nursing staff, if they're female to do all the—like, even brushing his teeth. Like, he could 100 per cent do it himself, but he wouldn't want to do it. And if you don't do it for him, well, then, he would say, I don't want to do it myself because you're supposed to serve me. (Senior Nurse 7_SA)

A few participants reported the importance of empowering the patients to seek assistance with oral care. Some patients were noted to experience a sense of vulnerability when it came to asking for help because of their culture:

They're very disempowered so giving patients permission to ask ‐politely—or ‘could you please just pass me my toothbrush and a bowl?’—we run hospitals like prisons and I think it's counterproductive. (Senior Nurse 5_ NSW)

### 3.2 Inadequate Knowledge and Education

The participants reported a lack of knowledge and educational preparedness on oral care and prevention of NV‐HAP. The subtheme constructed was a lack of knowledge and educational preparedness.

#### 3.2.1 Lack of Current Knowledge and Educational Preparedness

Most of the participants across the nursing workforce, junior nurses or the leadership nurses, from different states in Australia, with different qualifications, reported inadequate knowledge and education on oral care evidence‐based practice. They had minimal understanding of the relationship between oral care and NV‐HAP:

Overall, I'm probably not fantastic with my knowledge of things like that, just because my professional and research interests lie elsewhere. (Junior Nurse 2_VIC)

If I were to be honest, I wasn't 100 per cent wanting to trying to prevent non‐invasive ventilated‐associated pneumonia for these patients, but people might be subconsciously doing it and they did it and it actually prevent it from happening. (Senior Nurse 7_SA)

There were mixed views around teaching of fundamental skills. Some participants reported a lack of educational preparedness in current undergraduate and post‐graduate courses. Some nurses compared nursing skill sets for university trained and hospital trained nurses reporting a lack of certain nursing skill sets in university trained nurses:

And I'm not saying that university trained nurses don't have different skills, but I think those basic nursing skills are lacking and they don't think about those sorts of things as much as we do [hospital trained nurses]. (Junior Nurse 4_ACT)

Most participants reported that the focus in ICU education or training is more towards complex system assessment and management than fundamental nursing care:

It seems to be we teach systems [assessment of body systems]. We don't teach general hygiene. (Senior Nurse 1_VIC)

However, some participants reported that the lack of knowledge and understanding was not limited to nursing staff but other healthcare professionals as well:

So, not only have you got that lack of knowledge from nursing staff, that extends, from what I have seen in the intensive care contacts—context, I should say—it extends to the ICU doctors. (Senior Nurse 8_TAS)

### 3.3 Perception of Inadequate Use of Evidence‐Based Practice and Equipment

Most participants reported that there was no clear evidence‐based practice on oral care for non‐ventilated patients in ICU. Some participants reported that they used to have a unit routine for nursing care activities, which positively contributed to their adherence to oral care. Inconsistencies in oral care practices and assessments were reported by most of the nurses. Some participants reported that it was not their normal practice to undertake oral assessment as they do not have a tool or a guide to follow and there was no designated space for them to document it:

You won't see it though, in your head to toe assessment, there's no space for it at the back, when you're documenting and you're doing your neuro, CVS, respiratory, gut, you're doing all that, there's no separate title; you'll never see it, oral health assessment. You'll never see that though. (Senior Nurse 8_TAS)

This theme contained two subthemes: lack of policy or procedures and lack of consensus on preferred approaches and tools.

#### 3.3.1 Lack of Policy and Procedures

Most participants indicated that there were no policies, procedures, or guidelines for oral care in non‐ventilated patients in their units. There were no sources for reference to guide their practice:

We do probably have a lack of policy, I think, around that, but it always helps, and having that equipment. (Senior Nurse 9_ACT)

Some participants reported that they follow the ventilated patients two to four hourly policy. However, most of the participants reported relying on their personal preferences when providing oral care to patients, with some noting the practice of brushing the patient's teeth twice a day based on their individual choices:

—you just do what you know is the right thing to do when possible. (Senior Nurse 10_WA)

One participant reported having a guideline to use toothbrush and toothpaste at least three times a day on their non‐ventilated patients. The participant also mentioned that it was not consistent with dentists twice daily recommendations but those were their guidelines:

Our guideline says at least three times a day and particularly after meals while they're sick. Most dentists would not encourage that, they'd say twice a day. (Senior Nurse 5_NSW)

#### 3.3.2 Lack of Consensus on Preferred Approaches and Tools

Divergent opinions exist regarding oral care products and their accessibility. Some participants reported availability of a range of equipment, chlorhexidine mouthwash, toothbrushes, plain swabs. Some participants reported awareness of the available literature on increased patient mortality linked to the use of chlorhexidine mouthwash:

There's some evidence that chlorhexidine mouthwash might actually be harmful in terms of mortality, even though there may be an effect on [Hospital acquired pneumonia] HAP. (Senior Nurse 5_NSW)

Most of the participants from metropolitan tertiary ICUs expressed concerns regarding inadequate availability, specifically citing shortages of toothbrushes and oral care swabs. In these cases, some nurses depended on patients' personal supplies, meaning that if a patient did not bring any, they would be deprived of essential oral care:

We're just resource poor because resources are not prioritised to oral care. (Senior Nurse 8_TAS)

Most of the participants reported that there were no assessment tools for oral care, posing challenges in conducting oral assessment. Some participants drew parallels between pressure area care and oral care, highlighting the need for an oral care assessment tool:

We do not have an oral care assessment tool in our unit. Basically, we have an assessment of the patient, pressure area and all those other things, but there is definitely no tool for oral care assessment. (Senior Nurse 1_VIC)

They were not sure what was appropriate for them to assess or report and they all agreed an assessment tool will be helpful as a reminder and for documentation purposes.

### 3.4 Structured Support Needed for Evidence‐Based Practice

Most of the participants reported the need for supervision, reminders, feedback and support from multidisciplinary professionals and leaders to be able to effectively implement evidence‐based oral care for patients. There were three subthemes: leadership support, ongoing nurse and patient education and creating awareness of the importance of oral care.

#### 3.4.1 Leadership Support

Some participants highlighted the crucial need for support from both the nursing leadership team and medical staff. Concerns were expressed regarding some healthcare professionals not considering the oral care concerns raised by nurses, thus hindering the overall quality of patient care. One participant specifically mentioned:

–expectations from medical colleagues to assist in situations where assessing a patient's oral cavity proves challenging, emphasising the importance of collaborative problem‐solving for the benefit of patient well‐being. (Senior Nurse 5_NSW)

Furthermore, participants stressed the significance of support from the unit's leadership team regarding oral care. They reported the need for the team to provide essential resources and assistance when required:

need the awareness, need the resources, need the support and a bit of leadership to help them when too busy and stuff. (Junior Nurse 6_NSW)

#### 3.4.2 Ongoing Nurse and Patient Education

Most of the participants highlighted the critical role of education for healthcare professionals and patients alike on the importance of oral care. To address the challenge of ICU nurses attending formal education sessions, a few participants recommended continuous education through in‐services and bedside training. As one participant expressed:

Whether you're new to critical care or old to critical care, everyone can benefit from in‐services that improve their practice. So probably just ongoing education. (Senior Nurse 3_VIC)

Some participants proposed a planned approach to bedside education on oral care and NV‐HAP, encompassing both theoretical insights and practical demonstrations including patients as some patients refuse oral care. They suggested the inclusion of patients in these sessions to enhance relevance and applicability:

I always think bedside education is great because you don't have to gather people up to leave their bedsides. You can target two nurses at a time, discuss, demonstrate, and even involve patients in education like that. (Senior Nurse 10_WA)

#### 3.4.3 Create Awareness of the Importance of Oral Care

Some participants have drawn parallels between the routine swabbing of handwashing taps and sinks to assess their cleanliness and the imperative to gauge the prevalence of NV‐HAP. The participants expressed concern that NV‐HAP is not as widely discussed as VAP, which underscores their perception of the need for greater attention to NV‐HAP:

I suppose it [NV‐HAP] hasn't been talked about as much as it does with ventilated patients, but my understanding is that it can be just as prevalent. (Senior Nurse 3_VIC)

Some nurses emphasised the importance of raising awareness, drawing from their past encounter during the COVID‐19 pandemic:

I think we saw that [letting people know of the incidences helps] in the pandemic. (Senior Nurse 1_VIC)

The use of checklists, visual aids, such as pictures illustrating oral care practices or the consequences of poor oral care, auditing data, emerged as another recommended strategy:

It might encourage some of the non‐ventilated patients to go, Oh actually, it is really important. My nurse isn't just banging on about that for no reason. It is actually important. (Senior Nurse 10_WA)

We had X amount of patients in the last six months have it [hospital acquired pneumonia]', so it just potentially brings it more to the forefront for them. (Senior Nurse 9_ACT)

## 4 Discussion

This study explored intensive care nurses' clinical oral care practices in non‐ventilated patients. The findings demonstrate that participants understood the importance of oral care though it is not seen as a priority in day‐to‐day nursing care activities. The main barriers were lack of education and organisational factors such as lack of policies and procedures and lack of equipment. The participants suggested strategies to enhance the uptake of oral care in ICU: supervision and creating awareness of the importance of oral care in HAP prevention. Most of the barriers were reported across the ICUs and nursing workforce: senior and junior nurses.

Participants reported a lack of knowledge and education on current evidence‐based practices of oral care in non‐ventilated patients in ICU. These findings are consistent with similar studies undertaken in different countries [32, 33]. The evidence in this study and previous studies; nurses reported limited training in mouth care and the majority said they would like further training in oral care [33, 34, 35], According to previous studies educational training improved nurses' infection control behaviour; increased adherence with prevention strategies [36, 37]. This is consistent with Donabedian's model of Structure, Process and Outcome; where organisational factors and human resources such as healthcare professionals' education and their experience in ICU have an influence on the care they provide (process). The structural factors and processes within an organisation will also have an impact on the outcome, such as a lack of equipment, as evidenced in our study [38]. There is a need for oral care focused training and education or continuing education in oral care in ICU [33, 39, 40]. Participants in this study recommended involving patients and their families in oral care education and NV‐HAP prevention to empower patients and enhance adherence. This recommendation is supported by evidence from the Centers for Disease Control and Prevention (CDC), which states that education improves patient adherence to medication administration [41].

In this study, participants reported organisational barriers: lack of equipment, lack of policies, and need for leadership support. Study participants acknowledged that inadequate equipment (tooth brushes) was one of the barriers to providing oral care. The lack of equipment was associated with the low priority given to oral care in the context of all other patient care requirements. Previous studies have demonstrated that oral care is often given low priority among nursing activities in hospitals [33, 42]. However, most of the previous studies that reported a lack of equipment were from under‐resourced countries, such as Eritrea, South Africa and China [40, 43, 44]. This study is one of the first qualitative studies of barriers in oral care practices in ICU, providing insight into the lack of equipment in well‐resourced countries.

The lack of policies and procedures led to inconsistencies in oral care in non‐ventilated patients in ICU. These findings are consistent with previous studies [40]. According to Salamone and colleagues, the lack of protocols and policies on oral care demonstrates that oral care is given low priority in nursing in comparison to other activities [45]. According to Labeau and colleagues, the lack of policies and procedures on current evidence‐based practices might be related to healthcare workers' lack of knowledge, which is spread over all HAI prevention strategies [46]. The lack of policy and procedure might be linked to inconsistencies in nursing practice [23, 47]. As recommended by participants in this study, leadership support is required in policy and procedure development and ensuring enough equipment supply.

Absence of consensus on the best way to detect NV‐HAP might have led to a lack of auditing and surveillance of NV‐HAP in the participants' units. However, Bartley and colleagues reported that administrative coded data were 86% accurate in detecting NV‐HAP in an Australian healthcare setting [48]. In this study, participants recommended auditing and reporting of NV‐HAP cases and the frequency of oral activities to create awareness of the issue and current practices. These recommendations are consistent with the findings of a systematic review of 67 articles on promoting professional behaviour change in healthcare, which reported reminders, audit, feedback and educational outreach as key interventions to promote positive outcomes [49]. It is highly likely that the lack of auditing, reporting and reminders influenced oral care practices and NV‐HAP prevention in the participants' units.

## 5 Limitations

This study used purposive sampling and information power to inform sample size [27]. Although the participants were from across Australia, public, private, metropolitan and non‐metropolitan, we acknowledge that the views of these participants may not represent the views and experiences of all Australian intensive care nurses or ICU nurses in other countries. Most of the participants were experienced nurses with postgraduate qualification in ICU, which might not be a true representation of most ICUs.

## 6 Implications for Clinical Practice, Policy and Further Research

The findings from this study provide the following practical recommendations for clinical practice, policy and further research. Surveillance of NV‐HAP and auditing of oral care practices and reporting are recommended. There is a need for clear policy and procedures on oral care practices to prevent NV‐HAP. The findings indicated that participants prioritised oral care lower than other nursing care activities. They reported a lack of awareness of NV‐HAP cases or the link between oral care and NV‐HAP. There is a need for educational packages that focus on oral care and NV‐HAP prevention for healthcare professionals, patients and families. Future research is needed on exploring policymakers' and administrators attitudes towards oral care practices and NV‐HAP prevention in ICU.

## 7 Conclusion

Intensive care nurses are confronted with barriers to provide oral care, including inadequate education, lack of equipment and lack of support. These findings provide views that align with some previous studies conducted in different countries. This shows that oral care and prevention of NV‐HAP need focused attention in ICU to promote quality of patient care. Oral care is of paramount importance to NV‐HAP prevention and promotes patient comfort. It is therefore important that intensive care nurses have appropriate education, training, equipment, policies and procedures to provide evidence‐based oral care and promote patient safety and prevent NV‐HAP in ICU.

## Ethics Statement

This study received ethical approval from Monash University (Project ID: 37366) on March 7, 2023.

## Consent

The authors have nothing to report.

## Conflicts of Interest

The authors declare no conflicts of interest.

## Supporting information


**Supplementary File 1**: Interview questions.

## Data Availability

The data that support the findings of this study are available from the corresponding author upon reasonable request.
