# Supplementary material for: Nurses' Views on Barriers to Oral Care in Non‐Mechanically Ventilated Patients in the Intensive Care Unit: A Qualitative Study
Source: Nurs Crit Care. 2025 Sep 11;30(5):e70173. doi: 10.1111/nicc.70173 (PMC12424016; doi:10.1111/nicc.70173)
Supplement: Supplementary file 1 — Interview questions. [file NICC-30-0-s001.docx]

**Semi-structured interview questions**

1. Can you tell me your oral care practices in non-ventilated patients in ICU?
2. What’s your understanding of hospital acquired pneumonia (HAP) in non-ventilated patients in ICU?
3. Are you aware of any strategies used to prevent HAP in your unit? *(Probes: How often do you provide oral care; when do you complete a patient oral care assessment; time, diagnosis?*
4. What influences your oral care practices in non-ventilated patients in ICU? *(Probes: education, policy, current evidence? How?*
5. What factors stop you from performing oral care in non-ventilated patients in ICU?
6. What helps you to implement oral care in non-ventilated patients? (*Probe: patient condition, experience, title in ICU)*
7. Are there cultural considerations you have encountered when providing oral care?
8. Are there any oral practices in your unit that worries you?
9. How well does your unit adhere to prevention of HAP? (*Probe: Is there a focus in your unit on prevention of HAP in non-ventilated patients)*
10. What could you recommend to improve practice related to HAP prevention in non-ventilated patients in ICU?
11. Is there anything else you think would help us to understand HAP in prevention in your unit or in your practice?

**Thank you for your time.**
